# Supplementary material for: Mapping the role of tumor necrosis factor-related apoptosis-inducing ligand (TRAIL) and its receptors in chronic kidney disease: A scoping review protocol
Source: PLoS One. 2026 Apr 7;21(4):e0330796. doi: 10.1371/journal.pone.0330796 (PMC13056170; doi:10.1371/journal.pone.0330796)
Supplement: S1 File — (DOCX) [file pone.0330796.s001.docx]

**Supplementary file S1: Initial search strategies for each database**

**PubMed/Medline**

(((((((((((("tumor necrosis factor related apoptosis inducing ligand"[All Fields]) OR ("trail"[All Fields])) OR ("tnf related apoptosis inducing ligand"[All Fields])) OR ("apo2l"[All Fields])) OR ("apo 2l"[All Fields])) OR (Apo-2L)) OR ("tnfsf10"[All Fields])) OR ("tnfsf 10"[All Fields])) OR (TNFSF-10)) OR ("cd253"[All Fields])) OR ("cd 253"[All Fields])) OR (CD-253)) AND ((((((((((((("ckd"[All Fields]) OR ("chronic kidney disease"[All Fields])) OR ("ckdu"[All Fields])) OR ("chronic renal failure"[All Fields])) OR ("crf"[All Fields])) OR ("nephropathy"[All Fields])) OR ("glomerulonephritis"[All Fields])) OR ("polycystic kidney disease"[All Fields])) OR ("chronic interstitial nephritis"[All Fields])) OR ("chronic kidney failure"[All Fields])) OR (end-stage renal disease)) OR ("end stage renal disease"[All Fields])) OR ("esrd"[All Fields]))

**Scopus**

TITLE-ABS-KEY ( "Tumor necrosis factor related apoptosis inducing ligand" OR TRAIL OR "TNF related apoptosis inducing ligand" OR "APO 2L" OR APO2L OR "APO-2L" OR "TNFSF 10" OR TNFSF10 OR "TNFSF-10" OR "CD 253" OR CD253 OR "CD-253" ) AND TITLE-ABS-KEY ( CKD OR "chronic kidney disease" OR CKDu OR "chronic renal failure" OR CRF OR nephropathy OR glomerulonephritis OR "polycystic kidney disease" OR "chronic interstitial nephritis" OR "chronic kidney failure" OR "end-stage renal disease" OR "end stage renal disease" OR ESRD )

**EMBASE**

('tumor necrosis factor related apoptosis inducing ligand':ti,ab,kw OR trail:ti,ab,kw OR 'tnf related apoptosis inducing ligand':ti,ab,kw OR 'apo 2l':ti,ab,kw OR apo2l:ti,ab,kw OR 'apo-2l':ti,ab,kw OR 'tnfsf 10':ti,ab,kw OR tnfsf10:ti,ab,kw OR 'tnfsf-10':ti,ab,kw OR 'cd 253':ti,ab,kw OR cd253:ti,ab,kw OR 'cd-253':ti,ab,kw) AND (ckd:ti,ab,kw OR 'chronic kidney disease':ti,ab,kw OR ckdu:ti,ab,kw OR 'chronic renal failure':ti,ab,kw OR crf:ti,ab,kw OR nephropathy:ti,ab,kw OR glomerulonephritis:ti,ab,kw OR 'polycystic kidney disease':ti,ab,kw OR 'chronic interstitial nephritis':ti,ab,kw OR 'chronic kidney failure':ti,ab,kw OR 'end-stage renal disease':ti,ab,kw OR 'end stage renal disease':ti,ab,kw OR esrd:ti,ab,kw)

**Cochrane library**

("Tumor necrosis factor related apoptosis inducing ligand" OR TRAIL OR "TNF related apoptosis inducing ligand" OR "APO 2L" OR APO2L OR "APO-2L" OR "TNFSF 10" OR TNFSF10 OR "TNFSF-10" OR "CD 253" OR CD253 OR "CD-253"):ti,ab,kw AND (CKD OR "chronic kidney disease" OR CKDu OR "chronic renal failure" OR CRF OR nephropathy OR glomerulonephritis OR "polycystic kidney disease" OR "chronic interstitial nephritis" OR "chronic kidney failure" OR "end-stage renal disease" OR "end stage renal disease" OR ESRD):ti,ab,kw

**Science direct**

Title, abstract or author-specified keywords ("tumor necrosis factor related apoptosis inducing ligand" OR TRAIL OR "TNF related apoptosis inducing ligand" OR APO2L OR TNFSF10 OR CD253) AND (kidney disease OR Nephropathy OR renal failure)

**Web of Science**

TS=(("tumor necrosis factor related apoptosis inducing ligand" OR TRAIL OR "TNF related apoptosis inducing ligand" OR "APO 2L" OR APO2L OR "APO-2L" OR "TNFSF 10" OR TNFSF10 OR "TNFSF-10" OR "CD 253" OR CD253 OR "CD-253") AND (CKD OR "chronic kidney disease" OR CKDU OR "chronic renal failure" OR CRF OR nephropathy OR glomerulonephritis OR "polycystic kidney disease" OR "chronic interstitial nephritis" OR "chronic kidney failure" OR "end-stage renal disease" OR "end stage renal disease" OR ESRD))

**CINAHL**

TX(("tumor necrosis factor related apoptosis inducing ligand" OR TRAIL OR "TNF related apoptosis inducing ligand" OR "APO 2L" OR APO2L OR "APO-2L" OR "TNFSF 10" OR TNFSF10 OR "TNFSF-10" OR "CD 253" OR CD253 OR "CD-253") AND (CKD OR "chronic kidney disease" OR CKDU OR "chronic renal failure" OR CRF OR nephropathy OR glomerulonephritis OR "polycystic kidney disease" OR "chronic interstitial nephritis" OR "chronic kidney failure" OR "end-stage renal disease" OR "end stage renal disease" OR ESRD))

**Clinicaltrials.gov**

("Tumor necrosis factor related apoptosis inducing ligand" OR TRAIL OR "TNF related apoptosis inducing ligand" OR "APO 2L" OR APO2L OR "APO-2L" OR "TNFSF 10" OR TNFSF10 OR "TNFSF-10" OR "CD 253" OR CD253 OR "CD-253") AND (CKD OR "chronic kidney disease" OR CKDu OR "chronic renal failure" OR CRF OR nephropathy OR glomerulonephritis OR "polycystic kidney disease" OR "chronic interstitial nephritis" OR "chronic kidney failure" OR "end-stage renal disease" OR "end stage renal disease" OR ESRD)

**WHO ICTRP**

("Tumor necrosis factor related apoptosis inducing ligand" OR TRAIL OR "TNF related apoptosis inducing ligand" OR "APO 2L" OR APO2L OR "APO-2L" OR "TNFSF 10" OR TNFSF10 OR "TNFSF-10" OR "CD 253" OR CD253 OR "CD-253") AND (CKD OR "chronic kidney disease" OR CKDu OR "chronic renal failure" OR CRF OR nephropathy OR glomerulonephritis OR "polycystic kidney disease" OR "chronic interstitial nephritis" OR "chronic kidney failure" OR "end-stage renal disease" OR "end stage renal disease" OR ESRD)
