# Supplementary material for: Mapping the role of tumor necrosis factor-related apoptosis-inducing ligand (TRAIL) and its receptors in chronic kidney disease: A scoping review protocol
Source: PLoS One. 2026 Apr 7;21(4):e0330796. doi: 10.1371/journal.pone.0330796 (PMC13056170; doi:10.1371/journal.pone.0330796)
Supplement: Attachment — Submitted filename: Response to reviewers.pdf [file pone.0330796.s003.pdf]

Editors-in-chief,
PLOS One.

Dear Sir/Madam,

<u>Response to the reviewer's comments</u>

Thank you so much for reviewing the protocol article titled "The role of tumor necrosis factor-related apoptosis-inducing ligand (TRAIL) and its receptors in chronic kidney disease- a scoping review protocol. In response to the editors' and reviewers' comments, we have made the necessary revisions and explanations to address the concerns raised. A summary of our responses is attached to this letter.

Thank you for your time and consideration.

Sincerely,

Co- corresponding authors

Reviewer/Editor comments	Author comments	Edits as appeared in the review	Page Numbers
In the abstract, on page 2, line 21, it should be >10% globally, in the introduction line 47-48 also, please check and correct	Considering the comment given we have revised this information to present the most recent knowledge on the prevalence of CKD.	Chronic kidney disease (CKD) is increasingly recognized as a global public health concern, impacting about 700 million people worldwide.<br>Chronic kidney disease (CKD) is defined as kidney damage or glomerular filtration rate (GFR) <60 mL/min/1.73 m^2 for 3 months or more, irrespective of the cause (1) is a global health burden affecting more than 700 million people worldwide (2)	2,3
For Methodology, page number 5, for "methodological framework as described by Arksey and O'Malley and revisions made by the Joanna Briggs Institute (JBI)", instead of writing all stages in text, please provide the flow chart for the work described. This will be easy to follow and give more clarity and enhance the readability of the paper.	Added a figure	Figure 1 Methodological framework of the scoping review	6
On page number 13 for Stage 5: Collating and summarizing the results, data analysis is mentioned by several methods, adding few tests will be good for better	Revised	Data analysis will involve tabulating quantitative data and thematically analyzing qualitative data presented narratively or in tables. After contextualizing based on research questions, results of quantitative data analysis will be mainly presented using counts and frequencies, and proportions. The synthesis will be represented	14

understanding of correlation matrix		through tables, graphs, or charts, complemented by narrative descriptions.	
Line 28: "Joanna Bricks Institute" should be "Joanna Briggs Institute"	Revised	The proposed scoping review will be conducted in line with Arksey and O'Malley's methodological framework and the Joanna Briggs Institute (JBI) reviewer's manual	2
Line 79: Expand on previous research on TRAIL and receptors in CKD and outline knowledge gap and how your study fits into the current literature.	Expanded as suggested	Mechanistically, this ligand appears to influence tubulointerstitial injury, glomerular filtration, and vascular remodeling based on the receptor expression modulated by nature of cytotoxic insult and microenvironment. The balance between proapoptotic and antiapoptotic TRAIL receptors determines whether TRAIL signaling promotes cell death, survival, inflammation, or senescence of renal cells. For example, in early diabetic kidney disease (DKD) TRAIL has been associated with podocyte PANoptosis by expressing higher DR5 levels (15). Concurrently, metabolic stress and advanced glycation end products are shown to promote tubular cell senescence and maladaptive repair by expressing higher DcR2 levels (16,17). Likewise, the precise role of TRAIL and its receptors in CKDs of different etiologies remains under investigation and literature remains fragmented with limited synthesis of data	4
Line 82: State why a scoping review is a more appropriate approach than systematic review	Added a statement to highlight the appropriateness of the review approach	For this, we selected a scoping review approach since existing studies span across different experimental models, clinical observations, and molecular mechanisms among different CKD subtypes.	4, 5

Add a phrase to highlight the research purpose or thematic scope—e.g., mechanisms, clinical implications, or therapeutic targeting. • For instance: "Mapping the role of TRAIL and its receptors in chronic kidney disease: a scoping review protocol of inflammatory and apoptotic pathways"	Included	Mapping the role of tumor necrosis factor-related apoptosis-inducing ligand (TRAIL) and its receptors in chronic kidney disease- a scoping review protocol	1
What are the Objectives of this Study Protocol? State the overall objective and the specific objectives for this study protocol.	Included	The outcome review paper aims to summarize and highlight the existing research gaps related to TRAIL/TRAIL-R expression patterns, regulatory mechanisms and associations with clinical outcomes across different CKD etiologies. This scoping review protocol will define the transparent and systematic methodology to identify, chart and synthesize existing evidence.	5
What are the Rationale section for the protocol? The Authors should state the rationale or significance of this study protocol.	Rationale and the significance of the study included	Likewise, the precise role of TRAIL and its receptors in CKDs of different etiologies remains under investigation and literature remains fragmented with limited synthesis of data.<br>Therefore, mapping the existing evidence systematically will pave the way to identify the significance of TRAIL in CKD and thereby identify the areas that need to be further explored.	4,5
To enrich contextual depth, consider expanding on why TRAIL is biologically relevant to	Included	Mechanistically, this ligand appears to influence tubulointerstitial injury, glomerular filtration, and vascular remodeling based on the receptor expression	4

CKD pathology and highlighting gaps in current research:		modulated by nature of cytotoxic insult and microenvironment. The balance between proapoptotic and antiapoptotic TRAIL receptors determines whether TRAIL signaling promotes cell death, survival, inflammation, or senescence of renal cells. For example, in early diabetic kidney disease (DKD) TRAIL has been associated with podocyte PANoptosis by expressing higher DR5 levels (15). Concurrently, metabolic stress and advanced glycation end products are shown to promote tubular cell senescence and maladaptive repair by expressing higher DcR2 levels (16,17)	
In addition to outlining the five-stage framework, provide more detail on how extracted data will be categorized and interpreted. This signals analytical rigor beyond summary reporting:	Elaborated the data categorization and interpretation	Data will be charted using the data extraction framework (Table 2) that captures bibliographic details, study design, population characteristics, TRAIL related biomarkers, and outcomes in CKD progression. This will be conducted under 5 key subject areas, and this preliminary content may be amended as the review progresses.<br>1. Study characteristics<br>2. Population characteristics<br>3. TRAIL expression<br>4. Underlying mechanisms and regulatory pathways<br>5. Association with CKD progression	9
