# Supplementary material for: Mapping the role of tumor necrosis factor-related apoptosis-inducing ligand (TRAIL) and its receptors in chronic kidney disease: A scoping review protocol
Source: PLoS One. 2026 Apr 7;21(4):e0330796. doi: 10.1371/journal.pone.0330796 (PMC13056170; doi:10.1371/journal.pone.0330796)
Supplement: Attachment — Submitted filename: Response to reviewer.docx [file pone.0330796.s004.docx]

28^th^ January 2026

Editor-in-Chief

PLOS One.

Dear Editor,

We would like to thank the editorial team and reviewers for the time and effort invested in evaluating our manuscript titled ‘Mapping the role of tumor necrosis factor-related apoptosis-inducing ligand (TRAIL) and its receptors in chronic kidney disease: a scoping review protocol” (Manuscript ID: PONE-D-25-42046)’. We have carefully revised the manuscript to address all editorial and reviewer comments. All changes have been incorporated into the revised manuscript and herewith we have provided a point-by-point response to each comment.

Please note that we have updated our financial disclosure. The funding statement has been removed from the manuscript and is instead provided here, as requested. Updated funding statement is “This scoping review protocol is part of a major project supported by the Science and Technology Human Resource Development Project of the Ministry of Higher Education, Sri Lanka, funded by the Asian Development Bank (Grant No. R2RJ4).”

We all authors have read and approved the final manuscript and have no conflicts of interest to disclose. We confirm that this work is original and has not been published elsewhere, nor is it currently under consideration for publication elsewhere.

Thank you for considering our submission. We look forward to your response and the opportunity to publish in your esteemed journal.

Thank you

Sincerely,

Co-corresponding authors

| **Reviewer/Editor comments** | | **Response** | **Line no** |
| --- | --- | --- | --- |
| Journal requirements | | | |
| 1 | Please ensure that your manuscript meets PLOS ONE's style requirements, including those for file naming. The PLOS ONE style templates can be found at  <https://journals.plos.org/plosone/s/file?id=wjVg/PLOSOne_formatting_sample_main_body.pdf> and  [https://journals.plos.org/plosone/s/file?id=ba62/PLOSOne_formatting_sample_title_authors_affiliations.pdf](https://track.editorialmanager.com/CL0/https:%2F%2Fjournals.plos.org%2Fplosone%2Fs%2Ffile%3Fid=ba62%2FPLOSOne_formatting_sample_title_authors_affiliations.pdf/1/010f019b24e9cb5a-1e2a1db8-28d9-4f5b-80b9-b96184932f8a-000000/Y_maXew0f1GzX012e3VsUf1ek2Qwahpss1F8DMuFmmU=241) | Revised the manuscript according to the PLOS one journal style based on the given sample file. | - |
| 2 | Please note that funding information should not appear in any section or other areas of your manuscript. We will only publish funding information present in the Funding Statement section of the online submission form. Please remove any funding-related text from the manuscript | All funding related information has been removed from the manuscript text and is only available on the online submission form. | 194 |
| 3 | When completing the data availability statement of the submission form, you indicated that you will make your data available on acceptance. We strongly recommend all authors decide on a data sharing plan before acceptance, as the process can be lengthy and hold up publication timelines. Please note that, though access restrictions are acceptable now, your entire data will need to be made freely accessible if your manuscript is accepted for publication. This policy applies to all data except where public deposition would breach compliance with the protocol approved by your research ethics board. If you are unable to adhere to our open data policy, please kindly revise your statement to explain your reasoning and we will seek the editor's input on an exemption. Please be assured that, once you have provided your new statement, the assessment of your exemption will not hold up the peer review process | No datasets were generated or analysed during this scoping review protocol. All information required for the protocol is included in the manuscript and its supplementary materials. Data generated during the conduct of the scoping review will be made publicly available upon completion of the study. | - |
| 4 | Please note that funding information should not appear in any section or other areas of your manuscript. We will only publish funding information present in the Funding Statement section of the online submission form. Please remove any funding-related text from the manuscript. | All funding related information has been removed from the manuscript text and only available on the online submission form. | 194 |
| 5 | If the reviewer comments include a recommendation to cite specific previously published works, please review and evaluate these publications to determine whether they are relevant and should be cited. There is no requirement to cite these works unless the editor has indicated otherwise. | All recommended references were reviewed for relevance and cited. | 41-43 |
| Reviewer 1 | | | |
|  | Thank you for raising this search to identify potential new culprits implicated in CKD progression and identify gaps in knowledge. | We thank the reviewer for the positive and encouraging feedback. |  |
| Reviewer 2 | | | |
|  | /Database coverage is incomplete.  EMBASE and Web of Science should be added, as they capture much of the biomedical and pharmacological literature. Clinical trial registries (ClinicalTrials.gov, WHO ICTRP) and preprint servers (medRxiv, bioRxiv) should also be considered. | EMBASE and Web of Science were added to the electronic database search alongside PubMed/ MEDLINE, ScienceDirect, Scopus, CINAHL, and the Cochrane Library. Clinical trial registries (ClinicalTrials.gov and WHO ICTRP) will also be searched. Preprint servers are not included, as only peer-reviewed literature is considered. | 123 |
|  | /Incomplete search strategy reporting.  The manuscript provides only partial search terms. A full Boolean search string for each database (including synonyms such as TNFSF10, APO2L, CD253) should be added as a Supplementary File. | A complete and reproducible Boolean search strategy for each database has been provided as supplementary file 1. | 123-124 |
|  | /Language bias.  Restricting to English-only publications may exclude relevant non-English epidemiologic or regional studies. Consider including papers with English abstracts or at least acknowledging this limitation explicitly. | Considered including all studies without language bias and translating into English where needed. | 137 |
|  | /Clarify the purpose of MMAT quality assessment.  Scoping reviews typically do not exclude studies based on quality. Please clarify whether MMAT will be used descriptively (to summarize evidence quality) rather than for exclusion. | Clarified that MMAT will be used descriptively to summarize methodological quality. No studies will be excluded based on the MMAT scores. Lower quality studies (MMAT <50%) will be flagged and interpreted cautiously. | 159-160 |
|  | /Timeline inconsistency. The text states “articles published up to July 2025” but does not specify the actual search date. Indicate the exact date of the search (e.g., Search conducted on August 31, 2025). | Articles up to January 2026 will be included according to the current timeline. Indicated the exact date for article search as 1^st^ March 2026. | 123-125 |
|  | /Outcome definition unclear.  “Clinical outcomes” should be better defined (e.g., mortality, dialysis initiation, eGFR decline rate, cardiovascular events). Separate primary and secondary outcomes where possible. | Included mortality, dialysis initiation and cardiovascular events | Table2 (22) |
|  | /Assay variability not addressed.  Measurement of soluble TRAIL is highly method-dependent (different ELISA kits, storage conditions). Include assay type and sample source (plasma vs tissue) as data extraction items. | Sample source has been already included, and data extraction has been expanded to include assay type in the section of TRAIL expression. | Table 2 (15) |
| Reviewer 3 | | | |
|  | The PCC framework includes “all human studies, animal models, cell lines, and computational models.” Given the breadth of study types, how will the authors ensure a meaningful synthesis across such heterogeneous data? Will separate analyses or subgroup summaries be provided? | We clarified that data from heterogenous populations (human studies, animal models, cell lines, and related computational models) will be synthesized separately proving subgroup summaries. | 178-179 |
|  | Table 2 is detailed and appropriate. However, it may be beneficial to add a field for “type of CKD stage” (e.g., CKD stages 1-5, ESRD) under “Population characteristics” to facilitate staging-related analysis. Additionally, the authors mention thematic analysis for qualitative data. Will a specific framework (e.g., Braun & Clarke) be used? If so, please specify. | CKD stage has been added as a data extraction variable under population characteristics.  Qualitative findings will be analyzed using a descriptive approach, focusing on summarizing and mapping key characteristics and concepts reported in the included studies in line with the objectives of a scoping review. Therefore no specific thematic analysis will be performed. | Table 2 (9)  178-180 |
|  | The mixed-method appraisal tool (MMAT 2018) has been selected for quality appraisal, but the protocol states that “no studies will be excluded solely based on low scores.” How will low-quality studies (e.g., those with an MMAT score <50%) be handled in the synthesis? Will they be flagged, or will their findings be weighted differently to avoid misleading conclusions? | Clarified that MMAT will be used descriptively to summarize methodological quality. No studies will be excluded based on the MMAT scores. Lower quality studies (MMAT <50%) will be flagged and interpreted cautiously. | 182-184 |
|  | In the Introduction, the authors state: “Chronic kidney disease (CKD) is defined as kidney damage or glomerular filtration rate (GFR) <60 mL/min/1.73 m² for 3 months or more… affecting more than 700 million people worldwide.” The KDIGO 2021 update—which revises CKD staging and prevalence estimates—has not been cited. This outdated definition weakens the background. The protocol should reference the latest KDIGO guidelines (e.g., 2021 or 2024) for the definition of CKD. | Updated with KDIGO report 2024 as “Chronic kidney disease (CKD) is defined as “abnormalities of kidney structure or function, present for a minimum of 3 months, with implications for health” (1) is a global health burden affecting more than 800 million people worldwide (2).” | 41-42 |
|  | Please include a concept summary figure or graphical abstract to explain the entire implementation plan. | A conceptual summary figure illustrating the scope, methodology, and synthesis plan of the scoping review has been added. | Fig 1 |
|  | Abstract line 37: Rephrase “Google Scholar reference lists of review articles as grey literature sources” for clarity. Suggested revision: “Reference lists of relevant reviews retrieved from Google Scholar will also be screened for grey literature.” | Revised as suggested | 32-33 |
|  | Line 47-48: The definition of CKD is from 2005. Please consider citing more recent KDIGO guidelines (e.g., the 2024 update). | Most recent KDIGO guidelines were cited | 41-42 |
|  | Line 183-190 (Synthesis methods): Consider clarifying whether a meta-analysis is possible or if a narrative synthesis alone will be used | Consistent with the objectives of this scoping review, a meta-analysis will not be conducted. Results will be presented as narrative descriptions while including counts, frequencies, and proportions arise from quantitative data as appropriate. | 179-182 |
